# Copy number variations alter methylation and parallel *IGF2* overexpression in adrenal tumors

**DOI:** 10.1530/ERC-15-0086

**Published:** 2015-12

**Authors:** Helene Myrtue Nielsen, Alexandre How-Kit, Carole Guerin, Frederic Castinetti, Hans Kristian Moen Vollan, Catherine De Micco, Antoine Daunay, David Taieb, Peter Van Loo, Celine Besse, Vessela N Kristensen, Lise Lotte Hansen, Anne Barlier, Frederic Sebag, Jörg Tost

**Affiliations:** 1 Laboratory for Functional Genomics, Fondation Jean Dausset – Centre d'Etude du Polymorphisme Humain (CEPH), Paris, France; 2 Institute of Biomedicine, Aarhus University, Aarhus, Denmark; 3 Endocrine and Metabolic Surgery Department, AP-HM La Conception, Marseille, France; 4 Department of Endocrinology, AP-HM La Timone, Marseille, France; 5 Department of Genetics, Institute for Cancer Research, Oslo University Hospital, The Norwegian Radium Hospital, Oslo, Norway; 6 Division of Surgery, Transplantation and Cancer Medicine, Department of Oncology, Oslo University Hospital, Oslo, Norway; 7 The KG Jebsen Center for Breast Cancer Research, Institute for Clinical Medicine, Faculty of Medicine University of Oslo, Oslo, Norway; 8 Pathology Department, AP-HM La Timone, Marseille, France; 9 Nuclear Endocrine Imaging and Treatment Department, AP-HM La Timone, Marseille, France; 10 Cancer Research UK, London Research Institute, London, UK; 11 Department of Human Genetics, University of Leuven, Leuven, Belgium; 12 Genotyping Facilities, Centre National de Génotypage, CEA-Institut de Génomique, Evry, France; 13 Department of Clinical Molecular Biology (EpiGen), University of Oslo, Ahus Lokerod, Norway; 14 Laboratory of Molecular Biology, AP-HM La Conception and CRN2M, Aix-Marseille University, Marseille, France; 15 Laboratory for Epigenetics and Environment, Centre National de Génotypage, CEA-Institut de Génomique, Btiment G2, 2 rue Gaston Crmieux, CP 5721, Evry Cedex, 91057, France

**Keywords:** adrenocortical tumors, pheochromocytomas, IGF2, H19, cancer, imprinting, DNA methylation, copy number analysis, tetraploidy

## Abstract

Overexpression of *insulin growth factor 2* (*IGF2*) is a hallmark of adrenocortical carcinomas and pheochromocytomas. Previous studies investigating the *IGF2/H19* locus have mainly focused on a single molecular level such as genomic alterations or altered DNA methylation levels and the causal changes underlying *IGF2* overexpression are still not fully established. In the current study, we analyzed 62 tumors of the adrenal gland from patients with Conn's adenoma (CA, *n*=12), pheochromocytomas (PCC, *n*=10), adrenocortical benign tumors (ACBT, *n*=20), and adrenocortical carcinomas (ACC, *n*=20). Gene expression, somatic copy number variation of chr11p15.5, and DNA methylation status of three differential methylated regions of the *IGF2/H19* locus including the *H19* imprinting control region were integratively analyzed. *IGF2* overexpression was found in 85% of the ACCs and 100% of the PCCs compared to 23% observed in CAs and ACBTs. Copy number aberrations of chr11p15.5 were abundant in both PCCs and ACCs but while PCCs retained a diploid state, ACCs were frequently tetraploid (7/19). Loss of either a single allele or loss of two alleles of the same parental origin in tetraploid samples resulted in a uniparental disomy-like genotype. These copy number changes correlated with hypermethylation of the *H19* ICR suggesting that the lost alleles were the unmethylated maternal alleles. Our data provide conclusive evidence that loss of the maternal allele correlates with *IGF2* overexpression in adrenal tumors and that hypermethylation of the *H19* ICR is a consequence thereof.

## Introduction

Tumors developing in the adrenal gland include a heterogeneous group of adrenocortical and adrenomedullary malignancies (Brunt & Moley 2001, Grumbach *et al*. 2003). Most adrenal tumors are benign and non-hormone producing. However, adrenocortical masses require attention as hypersecretion of steroid hormones and metabolic changes may be present, which might lead to hypertension, abdominal pain, gender disorders, weight gain, or increased blood sugar levels depending on which adrenal hormone is dysregulated (Low & Sahi 2012, Hodin *et al*. 2014). Adrenal tumors with increased aldosterone secretion lead to Conn's syndrome (CA), whereas pheochromocytomas (PCCs) secrete excess amounts of catecholamines (Low & Sahi 2012, Hodin *et al*. 2014). Adrenocortical carcinomas (ACCs) can present as either non-functional or functional tumors and represent the most aggressive group of adrenal tumors. ACCs have often metastasized at the time of diagnosis and therefore are associated with poor prognosis (Volante *et al*. 2008).

Several genes and pathways have been implicated in the pathogenesis of ACCs including the WNT/β-Catenin pathway, and overexpression of the orphan nuclear receptor *SF1* (Fassnacht *et al*. 2011, Simon & Hammer 2012). Recently, integrated large-scale analyses have identified alterations in driver genes including *CTNNB1*, *TP53*, *CDKN2A*, *RB1*, *ZNRF3*, and *MEN1* (Barzon *et al*. 2001, Tissier *et al*. 2005, Assie *et al*. 2014, Juhlin *et al*. 2015). The insulin-like growth factor system has attracted much interest (Ribeiro & Latronico 2012) as the *Insulin-like Growth Factor 2* (*IGF2*) is the most frequently overexpressed gene in ACCs (de Fraipont *et al*. 2005). High levels of *IGF2* have been found to differentiate benign adrenocortical tumors from ACCs at both the RNA and the protein level (de Fraipont *et al*. 2005, Schmitt *et al*. 2006, Giordano *et al*. 2009, Ragazzon *et al*. 2011). Benign adrenal tumors express *IGF2* and *H19* at a level similar to normal adrenal tissue (Ilvesmaki *et al*. 1993*a*, Liu *et al*. 1995). In contrast, ACCs and PCCs show a downregulation of *H19* expression and overexpression of *IGF2* (Ilvesmaki *et al*. 1993*a*, Gicquel *et al*. 1997, Margetts *et al*. 2005, Slater *et al*. 2006, Soon *et al*. 2009, Meyer-Rochow *et al*. 2010, Ragazzon *et al*. 2011). Although IGF2 on its own is not sufficient for transformation, it has an active role in promoting ACC tumor growth (Guillaud-Bataille *et al*. 2014) and the IGF family plays an important role for the development of adrenal tumors, and has been proposed as therapeutic target (Ribeiro & Latronico 2012). *IGF2* and the neighboring *H19* are located on chromosome 11p15.5 and form the paradigmatic imprinted *IGF2/H19* locus (Supplementary Fig. 1, see section on supplementary data given at the end of this article). *IGF2* is expressed from the paternal allele only and due to its high expression levels in the fetal adrenal gland has been considered as a key mitogen for its early growth and development (Ilvesmaki *et al*. 1993*b*, Mesiano *et al*. 1993, O'Dell & Day 1998). After birth, *IGF2* expression levels decrease drastically and its expression is concentrated to the adrenal capsule and the periphery of the cortex (Baquedano *et al*. 2005). *H19* is maternally expressed and functions both as a long non-coding RNA involved in tumor suppression (Hao *et al*. 1993) and as a trans-regulator of a network of imprinted genes (Gabory *et al*. 2009).

Three differentially methylated regions (DMRs) are involved in the transcriptional regulation of the *IGF2/H19* locus (Supplementary Fig. 1, see section on supplementary data given at the end of this article). *IGF2* DMR0 and *IGF2* DMR2 are located between exons 2 and 3 and exons 8 and 9 respectively, while the *H19* DMR is located 4 kb upstream of the *H19* transcription start site. The *H19* DMR represents the imprinting control region (ICR) of the *IGF2/H19* locus and harbors seven binding sites for the methylation-sensitive insulator CTCF, a multifunctional protein involved in nuclear organization (Kanduri *et al*. 2000, Holmgren *et al*. 2001, Ohlsson *et al*. 2010), which brings downstream enhancers into physical proximity to either the *IGF2* or the *H19* promoter through parent-of-origin dependent methylation patterns of the *H19* ICR (Jinno *et al*. 1996, Frevel *et al*. 1999, Bell & Felsenfeld 2000, Kanduri *et al*. 2000).

The exact mechanisms underlying *IGF2* overexpression in adrenal tumors are poorly understood. DNA hypermethylation of the *H19* promoter region has previously been associated with *IGF2* overexpression in ACCs (Gao *et al*. 2002) as well as somatic copy number changes, where loss of the maternal allele was accompanied by a duplication of the paternal allele (Gicquel *et al*. 1994, 1997). Maternal loss of chr11p15.5 has also been implicated in the *IGF2* overexpression observed in PCCs (Margetts *et al*. 2005).

While genetic and epigenetic mechanisms are closely intertwined, it is currently unclear if DNA methylation and genetic aberrations are independent events leading to *IGF2* overexpression or if DNA methylation patterns reflect only changes at the genetic level. In the current study, we provide the first integrated analysis of gene expression, DNA methylation and genetic variation of the *IGF2/H19* locus in adrenal tumor subtypes using high-resolution molecular technologies to unravel the driving force behind *IGF2* overexpression in these tumors.

## Materials and methods

### Study group

Sixty-two adrenal tumor samples from the Timone Hospital (Marseille, France) were analyzed in this study. Clinical and pathological data are summarized in Table 1. Four groups of ACTs were included in the study: 12 aldosterone producing adenomas in the context of Conn's syndrome (CA), ten pheochromocytomas (PCCs), 20 adrenocortical carcinomas (ACCs) of which two samples (51 and 52) were derived from the two adrenal glands of the same patient, and 20 adrenocortical benign tumors (ACBT). ACBT were either non-secreting cortical tumors or cortisol secreting adrenal tumors with no evidence for malignancy. ACTH dependent bilateral adrenal hyperplasia and ACTH independent adrenal hyperplasia (primary pigmented nodular disease) were not included in this cohort. Adrenocortical tumors were staged using the Weiss score, PCCs were staged using the Pheochromocytoma of the Adrenal gland Scale Score (PASS), which both take histological features such as tumor size, presence of necrosis and mitotic activity including atypical mitoses into account (Weiss 1984, Thompson 2002, Lau & Weiss 2009). An adrenocortical tumor with a Weiss score of three or higher was classified as ACC and PCCs with a PASS score of four or higher were classified as malignant. All pheochromocytomas lacked a positive familial history indicating hereditary disease and had no mutations in the *SDHB*, *SDHD* and *VHL* genes as assessed by Sanger sequencing. Written informed consent was obtained from all patients and the study was approved by the local ethics committee.

### Isolation of DNA and RNA

All tumoral tissue samples were carefully evaluated by microscopy to ensure that sampling was from tumoral tissue and not adjacent normal tissue. Genomic DNA was isolated from 20 mg of fresh frozen tumors using the QIAamp DNA Mini Kit with a proteinase K treatment. Total RNA was extracted from 15 to 20 mg of the same tumor tissue using the RNeasy minikit (Qiagen) followed by DNAse treatment according to the manufacturer's instructions. DNA from whole blood samples of the corresponding patients were extracted using the MagAttract DNA Blood Midi M48 Kit (Qiagen).

### Gene expression analysis

According to the manufacturer's instructions, 500 ng of total RNA was reverse transcribed using Superscript III and oligo(dT) primers (Life Technologies). RNA concentrations were established using a NanoDrop 2000c spectrophotometer (Thermo Scientific, Wilmington, DE, USA) and the RNA integrity number (RIN) was assessed using an Agilent 2100 Bioanalyzer (Agilent Technologies, Massy, France).

Expression analyses were performed following the MIQE guidelines to ensure accurate quantification of gene expression (Bustin *et al*. 2009). The *IGF2* and *H19* expression assays used in this study are both spanning exon-exon boundaries and have previously been published (Dejeux *et al*. 2009, Koukoura *et al*. 2011) (Supplementary Table 1, see section on supplementary data given at the end of this article). Experiments were performed in duplicate for each sample using a cut-off of 10% coefficient of variance (CV). Two samples (9 and 16) were excluded from the expression analysis due to a CV value above the threshold and low RIN respectively. The average RIN among the remaining 60 RNA samples was 8.5. A dilution series of commercial cDNA (10–0.001 ng/μl) was prepared to determine PCR efficiency (*E*). For each primer set, *E* was >90%.

The geNorm reference gene selection kit (PrimerDesign, Southampton, UK) was used to identify the most stably expressed reference genes across the different adrenocortical tumors using the qbasePLUS Software (Biogazelle, Zwijnaarde, Belgium). The expression of *IGF2* and *H19* was normalized to the geometric mean of the three reference genes (*SDHA*, *ATP5B*, and *CYC1*). The final RT-qPCRs included 5 μl of cDNA (1 ng/μl), 5 μl primer (300 nM), and 10 μl of SYBR Green I Master mix (Roche). The real-time PCR program was initiated with 10 min at 95 °C followed by 45 cycles of 15 s at 95 °C and 60 s at 60 °C using a LightCycler 480 II (Roche). Melting curve analysis was performed after each run initiated with 5 s at 95 °C, 60 s at 65 °C and finally with a temperature ramping from 65 °C to 95 °C (0.1 °C/s) to exclude amplification of unspecific products.

### DNA methylation analysis by pyrosequencing

Quantitative DNA methylation values were obtained using pyrosequencing as previously described (Tost & Gut 2007). Briefly, 500 ng of genomic DNA was treated with sodium bisulfite using the EpiTect Bisulfite Kit (Qiagen) according to the manufacturer's instructions. The final PCR contained 10 ng of bisulfite treated DNA, 1×PCR buffer (Qiagen), assay specific MgCl_2_ concentrations, 100 μM of each nucleotide, 200 nM of each primer, and 2 U of HotStarTaq (Qiagen). The PCR program was initiated with an initial denaturation step of 15 min at 95 °C followed by 50 cycles of 30 s denaturation at 95 °C, 30 s at the assay specific annealing temperature and 15 s elongation at 72 °C. The final extension was performed for 5 min at 72 °C. Primer sequences and assay-specific modifications to the general protocol are given in Supplementary Table 1, see section on supplementary data given at the end of this article.

### Single nucleotide polymorphism analysis

Fifty-seven tumor samples had sufficient high-quality material available for genome-wide single nucleotide polymorphism (SNP) analysis using the IlluminaOmni2.5M array (Illumina, Inc., San Diego, CA, USA) (Gunderson *et al*. 2005, Peiffer *et al*. 2006)). Arrays were imaged using iScan scanners and the BeadStudio software (Illumina) was used to call genotypes and extract data for downstream analysis.

The log-transformed ratio between measured and expected SNP signal intensity (LogR) and B-allele frequencies (BAF) were visualized using tools implemented in the ‘copynumber’ R library (Gentleman *et al*. 2004, Nilsen *et al*. 2012). Inspection of raw data profiles demonstrated a previously described asymmetry in the intensity of the two alleles for each SNP remaining after the normalization steps implemented in Illumina's Genome Studio Software (Staaf *et al*. 2008). This affected both the allelic proportions and the copy number estimates. Data were corrected for this bias using a quantile normalization approach implemented in the tQN method (Staaf *et al*. 2008). Bias in the estimated copy number related to GC binding artifacts was corrected for using the method by (Cheng *et al*. 2011). Allele specific copy number estimates corrected for ploidy and infiltration of normal cells were identified using ASCAT (Van Loo *et al*. 2010). For the correct estimation of the percentage of the tumor cell fraction ASCAT is dependent on genomic aberrations. Therefore ASCAT cannot estimate the tumor percentage of diploid tumor samples without aberrations correctly. As such, we have excluded the tumor percentage estimation for diploid tumors without genetic aberrations in Table 2. Copy number changes were verified by genotyping rs680, which is located in *IGF2* (chr11:2,153,634) for paired tumor and blood samples heterozygous for rs680.

### Genotyping rs680

The PCR for genotyping contained 10 ng of genomic DNA, 1× PCR buffer (Qiagen), 1 mM MgSO_4_, 100 μM of each nucleotide, 200 nM of each primer, and 3 U of Platinum Taq DNA Polymerase High Fidelity (Life Technologies) in a final volume of 25 μl. The PCR program was initiated with a denaturation step of 4 min at 95 °C followed by 50 cycles of 30 s at 95 °C, an annealing temperature at 56 °C for 30 s, and elongation at 72 °C for 15 s. The final extension was performed for 4 min at 72 °C. Quantitative genotyping was performed on 10 μl of the PCR product using pyrosequencing. Primer sequences are given in Supplementary Table 1, see section on supplementary data given at the end of this article.

### Statistical analysis

The non-parametric Mann–Whitney test was used to assess potential differences between groups for the methylation and the expression analysis. A two-sided *t*-test was used to test for associations between categorized molecular alterations and categorical clinical parameters. Correlation analyses were made using the Spearman's rank correlation coefficient. A nominal *P* value <0.05 was considered significant.

## Results

To provide a comprehensive analysis of the mechanisms underlying *IGF2* overexpression in adrenal tumors, gene expression levels were integrated with copy number and ploidy analysis, and the DNA methylation status of three DMRs including the ICR upstream of *H19* (*H19* DMR) and two secondary DMRs (DMR0 and DMR2) within *IGF2* (Supplementary Fig. 1, see section on supplementary data given at the end of this article).

### Differential *IGF2* and *H19* expression characterize PCCs and ACCs

**Figure 1:**
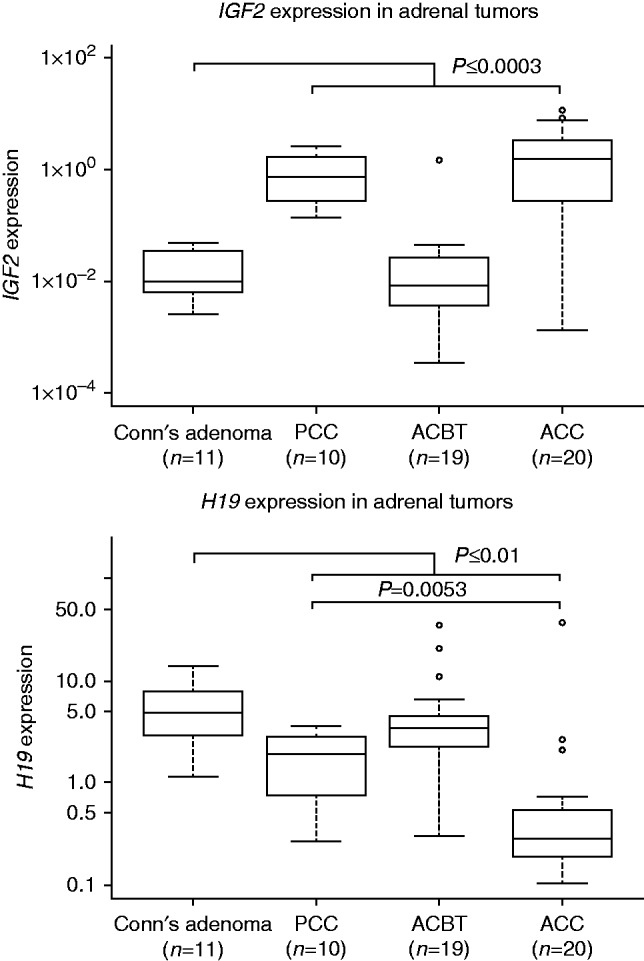
Quantitative expression analysis of *IGF2* and *H19* in adrenocortical tumors. *IGF2* and *H19* expression values are normalized to the geometric mean of the three reference genes (*SDHA*, *ATP5B*, and *CYC1*). Data is presented on a logarithmic scale.

Gene expression changes of *IGF2* and *H19* were analyzed in 60 fresh frozen adrenal tumors (Fig. 1). *IGF2* was considered overexpressed when the expression level was three times above the s.d. of the mean *IGF2* expression value observed for the CAs, which express *IGF2* and *H19* at levels comparable to normal adrenal tissue (Ilvesmaki *et al*. 1993*a*, Liu *et al*. 1995). *H19* was likewise considered downregulated when the expression level of individual samples was below three times the s.d. of the mean *H19* expression level observed for CAs.

*IGF2* showed overexpression in 85% (17/20) of the ACCs and 100% (10/10) of the PCCs, whereas only 4/19 ACBTs and 3/11 CAs showed overexpression of *IGF2* (*P*≤0.0003 for both ACC and PCC vs CAs; Mann–Whitney test) (Fig. 1). Expression levels did not correlate with clinical parameters such as presence of metastases, TNM stage. Only a tendency for a positive correlation between *IGF2* expression and tumor size for the ACCs was observed, but did not reach statistical significance (*R*^2^=0.273, *P*=0.067, Spearman's correlation). Decreased expression levels of *H19* also characterized both ACCs (18/20) and PCCs (7/10) when compared to the CA samples (*P*=0.0001 and *P*=0.01 respectively; Mann–Whitney test). However, the level of *H19* down regulation was less pronounced for PCCs compared to ACCs (*P*=0.0053; Mann–Whitney test) (Fig. 1). Concomitant *IGF2* overexpression and *H19* downregulation characterized the majority of the ACCs (15/20), PCCs (7/10), and a single ACBT, but was not present in all cases (Table 2).

### Aberrant DNA methylation is found throughout the *IGF2/H19* locus

**Figure 2:**
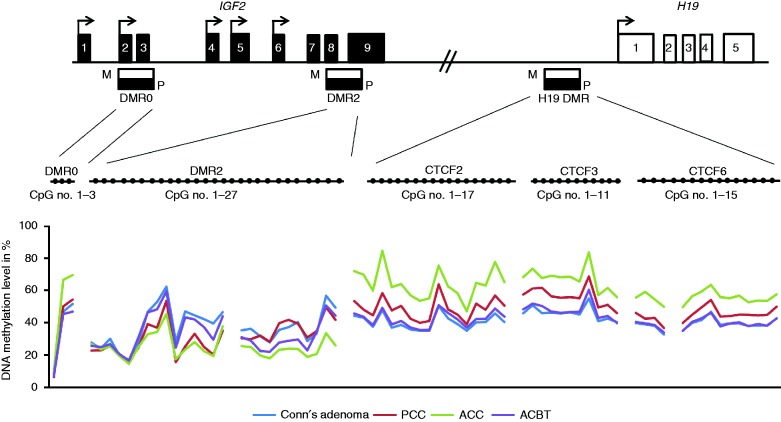
DNA methylation levels for the three DMRs of the *IGF2/H19* locus for each subgroup of adrenocortical tumors. (A) Schematic presentation of the *IGF2/H19* locus. *IGF2* harbors 9 exons whereas *H19* harbors five exons, which are indicated by the black and white squares. Transcription start sites are indicated with arrows. The *IGF2/H19* locus has three differential methylated regions (DMRs) named *DMR0*, *DMR2* and *H19* DMR. The parental-specific (M=maternal allele, P=paternal allele) DNA methylation status is presented by black bars for each DMR (black=methylated, white=unmethylated). The *H19* DMR makes up the imprinting control region (ICR) of the locus containing seven CTCF biding sites. For each DMR a number of CpG sites were analyzed (*DMR0*=3, *DMR2*=26, *CTCF2*=17, *CTCF3*=11, and for *CTCF6*=15) and the DNA methylation levels correlated highly between the three sites (*ρ*>0.75, *P*=2.2×10^−16^, Spearman's correlation). Each CpG site is presented as a filled circle. (B) The mean DNA methylation levels of single CpG sites are shown for each of the four adrenocortical tumor subtypes (Conn's adenoma, ACBTs, PCCs, and ACCs).

We further aimed to clarify the involvement of abnormal DNA methylation patterns of the three DMRs at the *IGF2/H19* locus (*IGF2* DMR0, *IGF2* DMR2, and *H19* DMR (ICR)) in adrenocortical tumorigenesis. For each analyzed region, a sample was scored as having aberrant DNA methylation if its mean DNA methylation level of the total number of CpG sites analyzed in each region differed by more than 10% from the CA samples. The median DNA methylation level for each CpG site analyzed is shown in Fig. 2 for each subgroup. The imprinting control region, *H19* ICR, contains seven CTCF binding sites, of which three were analyzed (CTCF2, CTCF3, and CTCF6), whereby the sixth binding site has been reported to correlate best with *IGF2* expression (Takai *et al*. 2001). The DNA methylation levels of the three CTCF binding sites analyzed were highly correlated (*ρ*>0.75, *P*=2.2×10^−16^, Spearman's correlation). PCCs and ACCs showed hypermethylation of the *H19* ICR compared to the samples from patients with CA and ACBTs (*P*<0.0001 for both the ACCs and PCCs, Mann–Whitney test). The DNA methylation level of the three CTCF binding sites was not as high for PCC samples as for ACCs (*P*=0.022, Mann–Whitney test) and this difference could neither be explained by copy number changes nor content of tumor cells in the samples. Of note, the *H19* ICR DNA methylation level correlated positively with the *IGF2* expression for the PCCs (*ρ*=0.93, *P*=0.00013, Spearman's correlation), while no correlation between *IGF2* expression and *H19* ICR methylation was observed for the ACCs (*ρ*=0.34, *P*=0.14, Spearman's correlation). Increased DNA methylation levels of the *H19* ICR were observed for four adenomas (samples 17, 19, 30, and 31). However, their DNA methylation levels only exceeded our chosen threshold by 1–5% and only one of these (sample 30) had concomitant *IGF2* overexpression.

A high correlation was also observed for the DNA methylation levels for the two regions analyzed within the *IGF2* DMR2 (DMR2a and DMR2b, *ρ*=0.63, *P*=1.43×10^−7^, Spearman's correlation). A gradient of hypomethylation of *IGF2* DMR2 was observed with CA samples presenting the highest DNA methylation level followed by the ACBTs with a significant difference between the two groups (*P*=0.0076, Mann–Whitney test). The PCCs and ACCs had the lowest DNA methylation levels when compared to CA samples (*P*=0.014 and *P*=0.019 respectively, Mann–Whitney test) (Fig. 2). The observed hypomethylation of the *IGF2* DMR2 was independent of the somatic copy number changes of the region (see the following section) and associated with neither Weiss nor PASS score nor *IGF2* expression levels (Table 2). DNA methylation changes did not correlate with any clinical parameters in the tumor groups.

The ACCs further displayed a slight, though significant, higher DNA methylation level for three CpG sites analyzed in the *IGF2* DMR0 compared to all other groups (*P*=0.0067, Kruskal–Wallis test), which correlated positively with the Weiss score (*ρ*=0.60, *P*=0.039, Spearman's correlation).

### Somatic copy number changes of chr11p15.5 are abundant in ACCs and PCCs

Genome-wide SNP analysis was used to establish the chromosomal status of chr11:1 986 296–2 223 233 harboring the *IGF2/H19* locus as this technology allows the detection of copy number changes as well as copy number neutral events. The chromosomal and allelic status was established using the BAF and Log R ratios, estimated by ASCAT (Van Loo *et al*. 2010), allowing discrimination between different forms of events: polyploidy, allelic loss and gain, and uniparental disomy (UPD). ASCAT results were confirmed by genotyping rs680 using pyrosequencing, which confirmed loss of heterozygosity for 8/8 informative samples (Supplementary Table 2, see section on supplementary data given at the end of this article).

**Figure 3:**
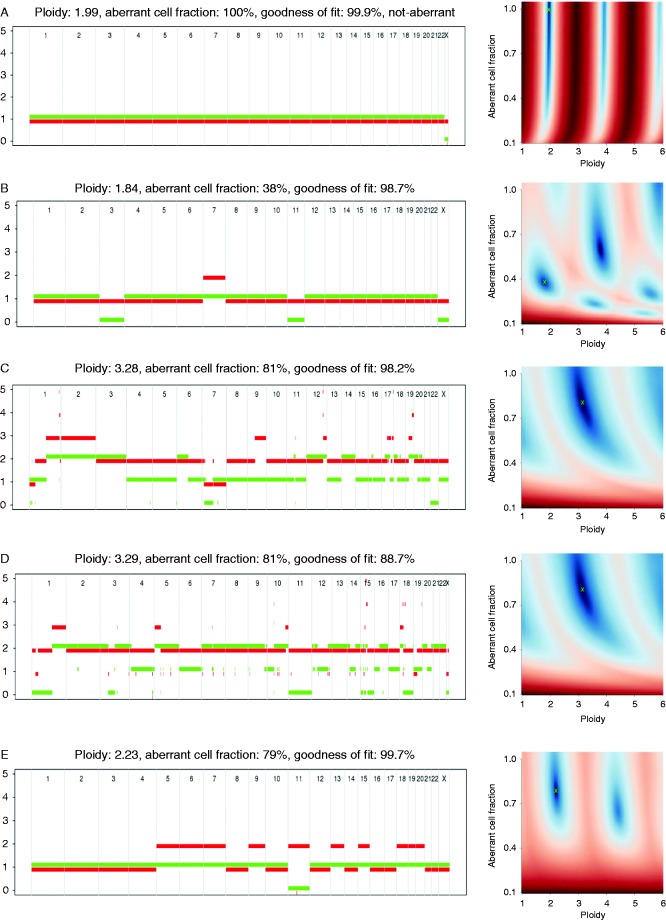
ASCAT profiles of adrenal tumors. (A) A Conn's adenoma sample (1) being diploid and with no somatic copy number changes at chr11p15.5 thus presenting a normal sample. (B) A tetraploid ACC sample (51), for which loss of a single allele resulted in triploidy of chr11p15. (C) An UPD-like genotype (57) being tetraploid with loss of two alleles of the same parental origin. (D) A diploid sample (39) with a copy number neutral variation presenting an UPD. (E) The PCC 33 represents samples being diploid and with loss of a single allele of chr11p15.

Somatic copy number changes of the *IGF2/H19* locus were restricted to PCC and ACC samples (Table 2). PCC samples remained in the diploid state, while ACCs were often tetraploid (7/19 analyzed) (Table 2). Representative samples showing different somatic copy number changes of the chr11p15.5 locus are shown in Fig. 3. Figure 3A represents a diploid sample with no genetic alterations accounting for 36/57 of the samples analyzed within this study including 12/12 CA samples, 16/16 ACBTs, 4/10 PCCs and 4/19 ACCs. Seven out of 12 diploid ACCs and half of the PCCs presented with loss of a single allele (Fig. 3B). Seven ACC samples were found to be tetraploid of which two samples (51 and 53) had lost a single allele of chr11p15.5 resulting in triploidy of this region (Fig. 3C). Another three tetraploid ACC samples (44, 46, and 57) presented with an UPD-like genotype as a consequence of loss of two alleles of the same parental origin (Fig. 3D). Of the two remaining tetraploid samples, ACC sample 61 had lost one of each allele, thus having normal allelic dosage, whereas the other (sample 62) did not show any loss of chr11p15.5. UPD is characterized by copy number neutral variation and occurred in a single ACC sample (45) and a single PCC sample (39), both of which were diploid (Fig. 3E).

### *IGF2* overexpression in PCCs and ACCs correlates with somatic copy number changes of chr11p15.5

**Figure 4:**
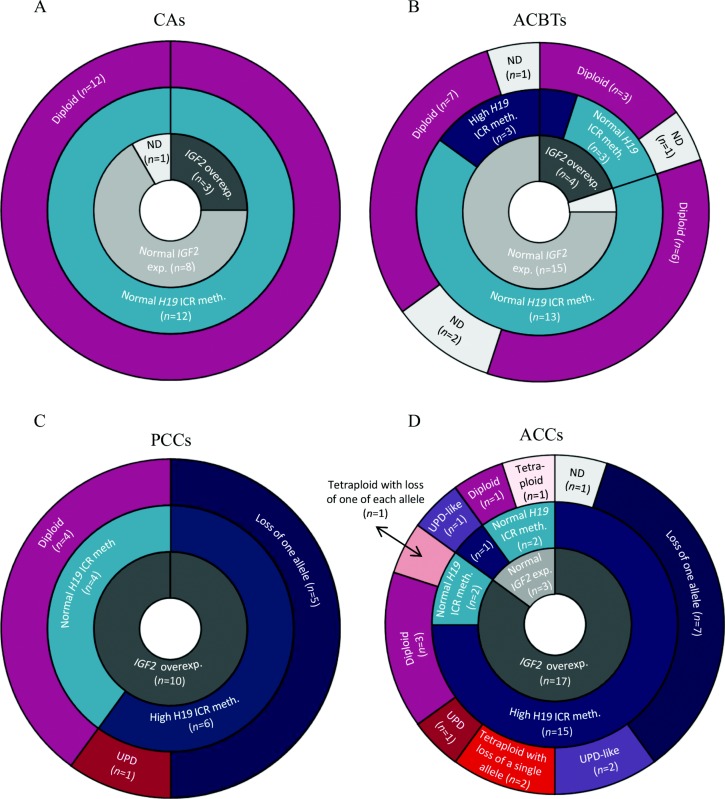
Doughnut diagrams integrating *IGF2* gene expression data with somatic copy number changes and DNA methylation alterations of the *IGF2/H19* locus. CAs and ACBTs were mainly characterized by normal *IGF2* expression, absence of copy number changes, and normal DNA methylation levels of the *H19* ICR. In contrast all PCCs showed overexpression of *IGF2*, where all samples with somatic copy number changes showed hypermethylation of the *H19* ICR. Seventeen out of 20 ACCs presented with overexpression of *IGF2* and this mainly correlated with somatic copy number changes of chr11p15.5 and hypermethylation of the *H19* ICR. Somatic copy number changes of chr11p15.5 were not exclusively associated with high *IGF2* expression levels as a ACC sample (57) with an UPD-like genotype had *IGF2* expression levels comparable to the samples from patients with Conn's adenoma.

Thirty-six diploid samples did not show any copy number changes at chr11p15.5 (Fig. 4), of which 13 (36%) overexpressed *IGF2* (Table 2). Of these 13 diploid samples, 11 samples had normal DNA methylation levels of the *H19* ICR (three CAs (2, 6, and 12), three ACBTs (14, 20, and 32), four PCCs (35, 38, 40, and 41), and a single ACC sample (48)). The two remaining samples with no copy number alterations and *IGF2* overexpression were diploid ACCs (50 and 54) and they both showed hypermethylation of the *H19* ICR. The two diploid samples (39 and 45) presenting with UPD both had high *IGF2* expression levels and high *H19* ICR methylation levels.

For the PCCs and ACCs, loss of a single allele in diploid samples was observed for 12/29 (41%) samples and therefore a recurrent event associated with *IGF2* overexpression and high *H19* ICR methylation levels (Fig. 4C and D).

Tetraploidy was a unique feature for ACCs in our study and with a frequency of 37% (7/19) it was a frequent event often accompanied by loss of either a single or two allele(s) of the same parental origin at chr11p15.5. Both tetraploid samples, which had lost a single allele and were therefore triploid for chr11p15.5 (51 and 53) had *IGF2* overexpressed and high *H19* ICR DNA methylation levels (Fig. 4D). Three tetraploid samples (44, 46, and 57) had lost two alleles of same parental origin resulting in an UPD-like genotype. Whereas the samples 44 and 46 showing an UPD-like genotype had *IGF2* overexpressed and high *H19* ICR methylation levels, sample 57 had *IGF2* expression levels equivalent to the CA samples, but still had the *H19* ICR hypermethylated (Fig. 4D) (Table 2). The tetraploid ACC sample 61 had lost one of each allele thus having normal allelic dosage accompanied by normal *H19* ICR DNA methylation levels and *IGF2* overexpression. The remaining tetraploid sample (62) presented with four allelic copies and was associated with normal *IGF2* expression levels and normal *H19* ICR methylation levels. The ACC samples 61 and 62 were the only tetraploid samples with equal number of parental allelic copies, which could explain the absence of high *H19* ICR DNA methylation levels.

Tumor samples with a higher number of paternal alleles compared to maternal ones had an increased probability of being metastatic (*P*<0.00001, *χ*^2^-test), but none of the other clinical parameters was associated to copy number changes or ploidy.

High DNA methylation levels of the imprinting control region were thus mainly observed for samples showing copy number changes of chr11p15.5 as 13/15 ACCs and 6/6 PCCs with copy number changes had increased DNA methylation levels (Fig. 4C and D) and the majority of adrenal tumors (90%) with somatic copy number changes also showed overexpression of *IGF2* (Fig. 4D).

## Discussion

ACCs and PCCs develop in the adrenal cortex and the adrenal medulla respectively, which are two distinct organs of separate embryonic origin. However, ACCs and PCCs share the characteristic *IGF2* overexpression. Unraveling the molecular mechanisms underlying *IGF2* overexpression is important as IGF2 contributes to cell proliferation and tumor progression (Livingstone 2013, Guillaud-Bataille *et al*. 2014). Studies investigating the underlying mechanisms of *IGF2* overexpression in adrenal tumors at more than a single molecular level are limited (Gicquel *et al*. 1994, 1997, Gao *et al*. 2002) and no study has so far integrated epigenetic and copy number variation obtained with quantitative high-resolution technologies with gene expression. In the present study we therefore integrated *IGF2* and *H19* expression levels with the DNA methylation levels of three regulatory DMRs located throughout the *IGF2/H19* locus and compared this to the copy number status and ploidy of chr11p15.5 in a cohort of 62 adrenal tumor samples.

In concordance with previous findings, we found a correlation between *IGF2* overexpression and the presence of malignant adrenocortical tumors (Ilvesmaki *et al*. 1993*a*, Gicquel *et al*. 1997, Slater *et al*. 2006, Soon *et al*. 2009, Ragazzon *et al*. 2011) as 85% of the ACCs overexpressed *IGF2*. However, similar to recently published data, the degree of overexpression did not correlate with the Weiss score (Guillaud-Bataille *et al*. 2014) or other clinical parameters. Moreover, *IGF2* overexpression was found in all PCCs, but here the degree of malignancy, as given by the PASS score, correlated with the level of overexpression, similar to previously published results (Margetts *et al*. 2005, Meyer-Rochow *et al*. 2010, Sandgren *et al*. 2010). While due to lack of normal tissue from the adrenal medulla, the gene expression level of the PCCs was compared to the expression level of adenoma in the context of Conn's Syndrome as reference and thus to a different tissue, the degree of the overexpression was probably not overestimated as the expression of *IGF2* in adults is restricted to the adrenal capsule and the periphery of the cortex (Baquedano *et al*. 2005), and the expression of *IGF2* in normal adrenal medullary cells is below the expression level in benign tumors (Meyer-Rochow *et al*. 2010). Furthermore, healthy adrenal medulla did not show IGF2 expression by immunohistochemical staining in human samples (Soon *et al*. 2009) or mouse models (Drelon *et al*. 2012), providing conclusive evidence that the detected high levels of *IGF2* in the PCCs are a tumor-specific alteration. Our data is further supported by the fact that the magnitude of overexpression detected in our study is very similar to a previously published study demonstrating the overexpression of *IGF2* in PCCs when compared to normal adrenal medulla (Waldmann *et al*. 2010).

*IGF2* overexpression has previously been associated with LOH at the *IGF2/H19* locus in adrenocortical tumor samples. 28/38 adrenal tumors with LOH of the *IGF2/H19* locus had lost the maternal allele and gained an extra copy of the paternal allele (Gicquel *et al*. 1994, 1997). In our cohort 15 out of 19 ACC samples analyzed had somatic copy number alterations at the *IGF2/H19* locus, with 6/15 samples having an extra copy of a single allele (Fig. 4D). As all six samples with UPD or an UPD-like genotype displayed hypermethylation of the *H19* ICR, we can assume that the unmethylated maternal allele is lost and accompanied by a duplication of the normally methylated paternal allele. However, it should be noted that no parental DNA was available to confirm this hypothesis. Due to the limitations of the method used in previous studies, the frequency of UPD cannot be easily compared as the ploidy of the tumors was not determined in the study of Gicquel *et al*. Even though they observed that loss of the maternal allele is accompanied by duplication of the paternal allele, it remains unknown whether this was due to an UPD in a diploid cell or loss of two alleles of the same parental origin in a tetraploid cell leading to an UPD-like genotype. As only a single ACC and a single PCC sample displayed UPD in our cohort, our data suggest that loss of the unmethylated maternal allele rather than UPD is the critical mechanism underlying *IGF2* overexpression. The presence of more paternal alleles than maternal alleles was significantly associated with the presence of metastases. However, the low number of metastatic samples in our study (*n*=4) makes it difficult to draw general conclusions from these results and will require validation in larger cohorts.

Tetraploidy was found to be a unique feature for the ACCs, which is supported by previous findings (Pignatelli *et al*. 1998, Blanes & Diaz-Cano 2006). In contrast to our study, tetraploidy has also been observed for PCCs using flow cytometry (Shono *et al*. 2002) and likewise has tetrasomy of single chromosomes been observed in samples of patients with CA (Shono *et al*. 2002), which also is in contrast to our and others (Lu *et al*. 1996). However, it cannot be ruled out that some PCCs could be tetraploid as only a limited number of tumors were analyzed in our study. It should be pointed out, that although the polyploidy of ACCs has previously been reported, none of the studies investigating the mechanism of *IGF2* overexpression took this information into account.

Gicquel *et al*. (1997) did not find *IGF2* overexpression restricted to samples with allelic imbalance of the *IGF2/H19* locus as 7/82 samples had *IGF2* overexpressed without any somatic copy number changes of chr11p15.5. This is in concordance with our findings as we found 13/36 diploid tumors overexpressed *IGF2*, of which 11 had normal *H19* ICR methylation levels. This indicates the involvement of an additional so far unknown mechanism leading to the characteristic overexpression of *IGF2* for PCCs and ACCs as well as for a subset of the ACBTs, which were found to be in general chromosomally stable in our study. The amplitude of the expression changes was not directly correlated to the different somatic copy number changes. A tetraploid sample with UPD-like genotype could present with a similar high expression as a diploid sample with no genetic alterations supporting again the hypothesis of other changes probably in trans influencing *IGF2* overexpression. In contrast to recently reported differences in DNA methylation patterns in the *H19* DMR between high and low IGF2 expressing ACCs (Guillaud-Bataille *et al*. 2014), we did not find any correlation between methylation patterns and expression levels of *IGF2* in the ACCs with their complex genetic background. However, a positive correlation was detected in the PCCs, which remained diploid.

One candidate transcription factor that could be interesting to investigate for its potential role in modulating *IGF2* expression levels is PLAG1 as its oncogenic function is mediated through the IGF2 pathway by binding to promoter 3 located between exon 4 and 5, resulting in *IGF2* overexpression (Hensen *et al*. 2002, Voz *et al*. 2004, Akhtar *et al*. 2012). Upstream enhancers have also been suggested to be involved in *IGF2* overexpression (Ulaner *et al*. 2003). However, whether the broad range from 4 to ∼600 fold of *IGF2* overexpression observed in the ACC group could be caused by transcription factors, upstream enhancers or other trans acting elements needs to be elucidated.

In conclusion, our data suggest that *IGF2* overexpression in adrenal tumors correlates mainly with allelic loss leading to an imbalance of the ratio between paternal and maternal alleles and that the aberrant DNA methylation levels observed for the *H19* ICR are a consequence thereof. Whether demethylation of the *IGF2* DMR2 contributes to overexpression of a subset of *IGF2* transcripts needs further investigation. Our study further underlines the importance to take ploidy into account to accurately discern the mechanism of gene-specific overexpression in tumors.

## Supplementary data

This is linked to the online version of the paper at http://dx.doi.org/10.1530/ERC-15-0086.

## Author contribution statement

H M Nielsen, A How-Kit, A Daunay and C Guerin performed laboratory experiments. H M Nielsen, A How-Kit, H K M Vollan, V N Kristensen, P V Loo, L L Hansen, J Tost were involved in the data analysis. C Guerin, F Castinetti, C D Micco, D Taieb, F Sebag and A Barlier were responsible for the patient cohorts, clinical and pathological analyses and sample preparation. H M Nielsen and J Tost provided the first draft of the manuscript. All authors participated in writing of the manuscript and approved the final version of the manuscript. A Barlier and J Tost initiated and designed the study. F Castinetti and C Guerin, and A Barlier and F Sebag contributed equally, and should be considered joint third and second last authors, respectively.

## Figures and Tables

**Table 1:** Clinical and pathological data on the adrenal tumors

**Patient subgroups**	**All**	**Percentage/range**
Conn's adenoma		
Number of samples	12	
Female	6	50%
Male	6	50%
Age at surgery, years (median, range)	47	(31–68)
Tumor size in mm (median, range)	14.5	(10–22)
Adrenocortical benign tumors (ACBT)		
Number of samples	20	
Female	16	80%
Male	4	20%
Age at surgery, years (median, range)	55.5	(23–68)
Weiss score (mean, range)	0.35	0–2
Tumor size in mm (median, range)	40	(20–60)
Pheochromocytomas (PCC)		
Number of samples	10	
Female	6	60%
Male	4	40%
Age at surgery, years (median, range)	50.5	(41–67)
PASS score (mean, range)	2.7	(0–9)
Tumor size in mm (median, range)	40	(26–80)
Malignant carcinomas (ACC)		
Number of samples	20	
Female	12	60%
Male	8	40%
Age at surgery, years (median, range)	48	(28–78)
Weiss score (mean, range)	5.65	(3–6)
No. of patients with metastases	4	
TNM stage I or II	7	
TNM stage III or IV	11	
Tumor size in mm (median, range)	50	(33–190)

**Table 2:** *IGF2* and *H19* expression levels compared to DNA methylation and genetic status of 11p15.5

Conn's adenoma									
ID	*IGF2* exp.	*H19* exp.	Genomic status of 11p15.5	Ploidy	*H19* ICR meth.	DMR2 meth.	DMR0 meth.		Tumor cell in %
1	Normal	Normal	Normal	2	Normal	Hypometh.	Normal		NA
2	Overexp.	Normal	Normal	2	Normal	Normal	Hypermeth.		NA
3	Normal	Normal	Normal	2	Normal	Hypometh.	Normal		NA
4	Normal	Normal	Normal	2	Normal	Normal	Hypermeth.		NA
5	Normal	Downreg.	Normal	2	Normal	Normal	Hypermeth.		NA
6	Overexp.	Normal	Normal	2	Normal	Normal	Normal		NA
7	Normal	Normal	Normal	2	Normal	Normal	Normal		NA
8	Normal	Downreg.	Normal	2	Normal	Normal	Normal		NA
9	NA	NA	Normal	2	Normal	Normal	Normal		NA
10	Normal	Normal	Normal	2	Normal	Hypometh.	Normal		NA
11	Normal	Normal	Normal	2	Normal	Hypometh.	NA		NA
12	Overexp.	Normal	Normal	2	Normal	Normal	Normal		NA
Adrenocortical Benign Tumours									
ID	*IGF2* exp.	*H19* exp.	Genomic status of 11p15.5	Ploidy	*H19* ICR meth.	DMR2 meth.	DMR0 meth.	Weiss	Tumor cell in %
13	Normal	Normal	Normal	2	Normal	Hypometh.	Normal	0	NA
14	Overexp.	Normal	Normal	2	Normal	Hypometh.	Hypermeth.	0	NA
15	Normal	Normal	Normal	2	Normal	Hypometh.	Normal	0	NA
16	NA	NA	Normal	2	Normal	Normal	Normal	0	NA
17	Normal	Downreg.	NA	NA	High	Hypometh.	Normal	0	NA
18	Normal	Normal	Normal	2	Normal	Normal	Normal	0	NA
19	Normal	Normal	Normal	2	High	Normal	Normal	0	NA
20	Overexp.	Downreg.	Normal	2	Normal	Hypometh.	Normal	0	NA
21	Normal	Normal	Normal	2	Normal	Hypometh.	Normal	0	NA
22	Normal	Downreg.	Normal	2	Normal	Normal	Normal	0	NA
23	Normal	Downreg.	Normal	2	Normal	Hypometh.	Normal	0	NA
24	Normal	Normal	Normal	2	Normal	Hypometh.	Normal	1	NA
25	Normal	Normal	Normal	2	Normal	Normal	Normal	0	NA
26	Normal	Normal	NA	NA	Normal	Normal	Normal	2	NA
27	Normal	Downreg.	Normal	2	Normal	Hypometh.	Normal	2	NA
28	Normal	Normal	Normal	2	Normal	Hypometh.	Normal	0	NA
29	Normal	Normal	NA	NA	Normal	Hypometh.	Normal	0	NA
30	Overexp.	Normal	NA	NA	High	Hypometh.	Hypermeth.	0	NA
31	Normal	Downreg.	Normal	2	High	Hypometh.	Normal	0	NA
32	Overexp.	Normal	Normal	2	Normal	Normal	Normal	0	NA
Pheochromocytomas									
ID	*IGF2* exp.	*H19* exp.	Copy number status of 11p15.5	Ploidy	*H19* ICR meth.	DMR2 meth.	DMR0 meth.	PASS	Tumor cell in %
33	Overexp.	Downreg.	Loss of one allele	2	High	Normal	Normal	2	38%
34	Overexp.	Downreg.	Loss of one allele	2	High	Hypometh.	Normal	6	53%
35	Overexp.	Normal	Normal	2	Normal	Normal	Hypermeth.	6	38%
36	Overexp.	Downreg.	Loss of one allele	2	High	Hypometh.	Normal	1	53%
37	Overexp.	Downreg.	Normal	2	Normal	Hypometh.	Normal	0	71%
38	Overexp.	Normal	Loss of one allele	2	High	Hypometh.	Hypermeth.	9	60%
39	Overexp.	Downreg.	UPD	2	High	Hypometh.	Hypermeth.	0	79%
40	Overexp.	Normal	Normal	2	Normal	Hypometh.	Normal	0	65%
41	Overexp.	Downreg.	Normal	2	Normal	Hypometh.	Normal	0	81%
42	Overexp.	Downreg.	Loss of one allele	2	High	Hypometh.	Normal	3	NA
Adrenocortical Carcinoma									
ID	*IGF2* exp.	*H19* exp.	Copy number status of 11p15.5	Ploidy	*H19* ICR meth.	DMR2 meth.	DMR0 meth.	Weiss	Tumor cell in %
43	Overexp.	Downreg.	Loss of one allele	2	High	Hypometh.	Hypermeth.	3	54%
44	Overexp.	Downreg.	UPD genotype	4	High	Hypometh.	Hypermeth.	7	72%
45	Overexp.	Downreg.	UPD	2	High	Hypometh.	Normal	3	70%
46	Overexp.	Downreg.	UPD genotype	4	High	Hypometh.	Hypermeth.	5	86%
47	Overexp.	Downreg.	Loss of one allele	2	High	Hypometh.	Normal	4	84%
48	Overexp.	Normal	Normal	2	Normal	Normal	Normal	3	59%
49	Overexp.	Normal	Loss of one allele	2	High	Hypometh.	Hypermeth.	5	68%
50	Overexp.	Downreg.	Normal	2	High	Normal	Hypermeth.	8	NA
51	Overexp.	Downreg.	Triploid	4	High	Hypometh.	Hypermeth.	8	81%
52	Overexp.	Downreg.	NA	NA	High	Hypometh.	Hypermeth.	8	NA
53	Overexp.	Downreg.	Triploid	4	High	Normal	Hypermeth.	6	32%
54	Overexp.	Downreg.	Normal	2	High	Normal	Hypermeth.	6	95%
55	Overexp.	Downreg.	Loss of one allele	2	High	Hypometh.	Hypermeth.	8	88%
56	Overexp.	Downreg.	Loss of one allele	2	High	Hypometh.	Normal	2	72%
57	Normal	Downreg.	UPD genotype	4	High	Normal	Hypermeth.	8	81%
58	Normal	Downreg.	Normal	2	Normal	Normal	Normal	7	39%
59	Overexp.	Downreg.	Loss of one allele	2	High	Hypometh.	Hypermeth.	6	70%
60	Overexp.	Downreg.	Loss of one allele	2	High	Hypometh.	Hypermeth.	7	67%
61	Overexp.	Downreg.	Loss of two alleles (one of each)	4	Normal	Hypometh.	Hypermeth.	5	59%
62	Normal	Downreg.	Tetraploid	4	Normal	Hypometh.	Hypometh.	4	81%

NA, no aberrations; for correct estimation of the tumor percentage, ASCAT is dependent on genomic aberrations. For diploid tumors without aberrations, the tumor percentage can therefore not be calculated.

## References

[bib1] Akhtar M, Holmgren C, Gondor A, Vesterlund M, Kanduri C, Larsson C, Ekstrom TJ (2012). Cell type and context-specific function of PLAG1 for IGF2 P3 promoter activity. International Journal of Oncology.

[bib2] Assie G, Letouze E, Fassnacht M, Jouinot A, Luscap W, Barreau O, Omeiri H, Rodriguez S, Perlemoine K, Rene-Corail F (2014). Integrated genomic characterization of adrenocortical carcinoma. Nature Genetics.

[bib3] Baquedano MS, Berensztein E, Saraco N, Dorn GV, de Davila MT, Rivarola MA, Belgorosky A (2005). Expression of the IGF system in human adrenal tissues from early infancy to late puberty: implications for the development of adrenarche. Pediatric Research.

[bib4] Barzon L, Chilosi M, Fallo F, Martignoni G, Montagna L, Palu G, Boscaro M (2001). Molecular analysis of CDKN1C and TP53 in sporadic adrenal tumors. European Journal of Endocrinology.

[bib5] Bell AC, Felsenfeld G (2000). Methylation of a CTCF-dependent boundary controls imprinted expression of the Igf2 gene. Nature.

[bib6] Blanes A, Diaz-Cano SJ (2006). DNA and kinetic heterogeneity during the clonal evolution of adrenocortical proliferative lesions. Human Pathology.

[bib7] Brunt LM, Moley JF (2001). Adrenal incidentaloma. World Journal of Surgery.

[bib8] Bustin SA, Benes V, Garson JA, Hellemans J, Huggett J, Kubista M, Mueller R, Nolan T, Pfaffl MW, Shipley GL (2009). The MIQE guidelines: minimum information for publication of quantitative real-time PCR experiments. Clinical Chemistry.

[bib9] Cheng J, Vanneste E, Konings P, Voet T, Vermeesch JR, Moreau Y (2011). Single-cell copy number variation detection. Genome Biology.

[bib10] Dejeux E, Olaso R, Dousset B, Audebourg A, Gut IG, Terris B, Tost J (2009). Hypermethylation of the IGF2 differentially methylated region 2 is a specific event in insulinomas leading to loss-of-imprinting and overexpression. Endocrine-Related Cancer.

[bib11] Drelon C, Berthon A, Ragazzon B, Tissier F, Bandiera R, Sahut-Barnola I, de Joussineau C, Batisse-Lignier M, Lefrancois-Martinez AM, Bertherat J (2012). Analysis of the role of Igf2 in adrenal tumour development in transgenic mouse models. PLoS ONE.

[bib12] Fassnacht M, Libe R, Kroiss M, Allolio B (2011). Adrenocortical carcinoma: a clinician's update. Nature Reviews. Endocrinology.

[bib13] de Fraipont F, El Atifi M, Cherradi N, Le Moigne G, Defaye G, Houlgatte R, Bertherat J, Bertagna X, Plouin PF, Baudin E (2005). Gene expression profiling of human adrenocortical tumors using complementary deoxyribonucleic Acid microarrays identifies several candidate genes as markers of malignancy. Journal of Clinical Endocrinology and Metabolism.

[bib14] Frevel MA, Hornberg JJ, Reeve AE (1999). A potential imprint control element: identification of a conserved 42 bp sequence upstream of H19. Trends in Genetics.

[bib15] Gabory A, Ripoche MA, Le Digarcher A, Watrin F, Ziyyat A, Forne T, Jammes H, Ainscough JF, Surani MA, Journot L (2009). H19 acts as a trans regulator of the imprinted gene network controlling growth in mice. Development.

[bib16] Gao ZH, Suppola S, Liu J, Heikkila P, Janne J, Voutilainen R (2002). Association of H19 promoter methylation with the expression of H19 and IGF-II genes in adrenocortical tumors. Journal of Clinical Endocrinology and Metabolism.

[bib17] Gentleman RC, Carey VJ, Bates DM, Bolstad B, Dettling M, Dudoit S, Ellis B, Gautier L, Ge Y, Gentry J (2004). Bioconductor: open software development for computational biology and bioinformatics. Genome Biology.

[bib18] Gicquel C, Bertagna X, Schneid H, Francillard-Leblond M, Luton JP, Girard F, Le Bouc Y (1994). Rearrangements at the 11p15 locus and overexpression of insulin-like growth factor-II gene in sporadic adrenocortical tumors. Journal of Clinical Endocrinology and Metabolism.

[bib19] Gicquel C, Raffin-Sanson ML, Gaston V, Bertagna X, Plouin PF, Schlumberger M, Louvel A, Luton JP, Le Bouc Y (1997). Structural and functional abnormalities at 11p15 are associated with the malignant phenotype in sporadic adrenocortical tumors: study on a series of 82 tumors. Journal of Clinical Endocrinology and Metabolism.

[bib20] Giordano TJ, Kuick R, Else T, Gauger PG, Vinco M, Bauersfeld J, Sanders D, Thomas DG, Doherty G, Hammer G (2009). Molecular classification and prognostication of adrenocortical tumors by transcriptome profiling. Clinical Cancer Research.

[bib21] Grumbach MM, Biller BM, Braunstein GD, Campbell KK, Carney JA, Godley PA, Harris EL, Lee JK, Oertel YC, Posner MC (2003). Management of the clinically inapparent adrenal mass ("incidentaloma"). Annals of Internal Medicine.

[bib22] Guillaud-Bataille M, Ragazzon B, de Reynies A, Chevalier C, Francillard I, Barreau O, Steunou V, Guillemot J, Tissier F, Rizk-Rabin M (2014). IGF2 Promotes growth of adrenocortical carcinoma cells, but its overexpression does not modify phenotypic and molecular features of adrenocortical carcinoma. PLoS ONE.

[bib23] Gunderson KL, Steemers FJ, Lee G, Mendoza LG, Chee MS (2005). A genome-wide scalable SNP genotyping assay using microarray technology. Nature Genetics.

[bib24] Hao Y, Crenshaw T, Moulton T, Newcomb E, Tycko B (1993). Tumour-suppressor activity of H19 RNA. Nature.

[bib25] Hensen K, Van Valckenborgh IC, Kas K, Van de Ven WJ, Voz ML (2002). The tumorigenic diversity of the three PLAG family members is associated with different DNA binding capacities. Cancer Research.

[bib26] Hodin R, Lubitz C, Phitayakorn R, Stephen A (2014). Diagnosis and management of pheochromocytoma. Current Problems in Surgery.

[bib27] Holmgren C, Kanduri C, Dell G, Ward A, Mukhopadhya R, Kanduri M, Lobanenkov V, Ohlsson R (2001). CpG methylation regulates the Igf2/H19 insulator. Current Biology.

[bib28] Ilvesmaki V, Kahri AI, Miettinen PJ, Voutilainen R (1993a). Insulin-like growth factors (IGFs) and their receptors in adrenal tumors: high IGF-II expression in functional adrenocortical carcinomas. Journal of Clinical Endocrinology and Metabolism.

[bib29] Ilvesmaki V, Blum WF, Voutilainen R (1993b). Insulin-like growth factor-II in human fetal adrenals: regulation by ACTH, protein kinase C and growth factors. Journal of Endocrinology.

[bib30] Jinno Y, Sengoku K, Nakao M, Tamate K, Miyamoto T, Matsuzaka T, Sutcliffe JS, Anan T, Takuma N, Nishiwaki K (1996). Mouse/human sequence divergence in a region with a paternal-specific methylation imprint at the human H19 locus. Human Molecular Genetics.

[bib31] Juhlin CC, Goh G, Healy JM, Fonseca AL, Scholl UI, Stenman A, Kunstman JW, Brown TC, Overton JD, Mane SM (2015). Whole-exome sequencing characterizes the landscape of somatic mutations and copy number alterations in adrenocortical carcinoma. Journal of Clinical Endocrinology and Metabolism.

[bib32] Kanduri C, Pant V, Loukinov D, Pugacheva E, Qi CF, Wolffe A, Ohlsson R, Lobanenkov VV (2000). Functional association of CTCF with the insulator upstream of the H19 gene is parent of origin-specific and methylation-sensitive. Current Biology.

[bib33] Koukoura O, Sifakis S, Zaravinos A, Apostolidou S, Jones A, Hajiioannou J, Widschwendter M, Spandidos DA (2011). Hypomethylation along with increased H19 expression in placentas from pregnancies complicated with fetal growth restriction. Placenta.

[bib34] Lau SK, Weiss LM (2009). The Weiss system for evaluating adrenocortical neoplasms: 25 years later. Human Pathology.

[bib35] Liu J, Kahri AI, Heikkila P, Ilvesmaki V, Voutilainen R (1995). H19 and insulin-like growth factor-II gene expression in adrenal tumors and cultured adrenal cells. Journal of Clinical Endocrinology and Metabolism.

[bib36] Livingstone C (2013). IGF2 and cancer. Endocrine-Related Cancer.

[bib37] Low G, Sahi K (2012). Clinical and imaging overview of functional adrenal neoplasms. International Journal of Urology.

[bib38] Lu X, Stallmach T, Gebbers JO (1996). Image cytometric DNA analysis of adrenocortical neoplasms as a prognostic parameter: a clinico-pathologic study of 13 patients. Analytical Cellular Pathology.

[bib39] Margetts CD, Astuti D, Gentle DC, Cooper WN, Cascon A, Catchpoole D, Robledo M, Neumann HP, Latif F, Maher ER (2005). Epigenetic analysis of HIC1, CASP8, FLIP, TSP1, DCR1, DCR2, DR4, DR5, KvDMR1, H19 and preferential 11p15.5 maternal-allele loss in von Hippel-Lindau and sporadic phaeochromocytomas. Endocrine-Related Cancer.

[bib40] Mesiano S, Mellon SH, Jaffe RB (1993). Mitogenic action, regulation, and localization of insulin-like growth factors in the human fetal adrenal gland. Journal of Clinical Endocrinology and Metabolism.

[bib41] Meyer-Rochow GY, Jackson NE, Conaglen JV, Whittle DE, Kunnimalaiyaan M, Chen H, Westin G, Sandgren J, Stalberg P, Khanafshar E (2010). MicroRNA profiling of benign and malignant pheochromocytomas identifies novel diagnostic and therapeutic targets. Endocrine-Related Cancer.

[bib42] Nilsen G, Liestol K, Van Loo P, Moen Vollan HK, Eide MB, Rueda OM, Chin SF, Russell R, Baumbusch LO, Caldas C (2012). Copynumber: efficient algorithms for single- and multi-track copy number segmentation. BMC Genomics.

[bib43] O'Dell SD, Day IN (1998). Insulin-like growth factor II (IGF-II). International Journal of Biochemistry & Cell Biology.

[bib44] Ohlsson R, Lobanenkov V, Klenova E (2010). Does CTCF mediate between nuclear organization and gene expression?. BioEssays.

[bib45] Peiffer DA, Le JM, Steemers FJ, Chang W, Jenniges T, Garcia F, Haden K, Li J, Shaw CA, Belmont J (2006). High-resolution genomic profiling of chromosomal aberrations using Infinium whole-genome genotyping. Genome Research.

[bib46] Pignatelli D, Leitao D, Maia M, Schmidt F (1998). DNA quantification and ploidy patterns in human adrenocortical neoplasms. Endocrine Research.

[bib47] Ragazzon B, Assie G, Bertherat J (2011). Transcriptome analysis of adrenocortical cancers: from molecular classification to the identification of new treatments. Endocrine-Related Cancer.

[bib48] Ribeiro TC, Latronico AC (2012). Insulin-like growth factor system on adrenocortical tumorigenesis. Molecular and Cellular Endocrinology.

[bib49] Sandgren J, Andersson R, Rada-Iglesias A, Enroth S, Akerstrom G, Dumanski JP, Komorowski J, Westin G, Wadelius C (2010). Integrative epigenomic and genomic analysis of malignant pheochromocytoma. Experimental & Molecular Medicine.

[bib50] Schmitt A, Saremaslani P, Schmid S, Rousson V, Montani M, Schmid DM, Heitz PU, Komminoth P, Perren A (2006). IGFII and MIB1 immunohistochemistry is helpful for the differentiation of benign from malignant adrenocortical tumours. Histopathology.

[bib51] Shono T, Sakai H, Takehara K, Honda S, Kanetake H (2002). Analysis of numerical chromosomal aberrations in adrenal cortical neoplasms by fluorescence in situ hybridization. Journal of Urology.

[bib52] Simon DP, Hammer GD (2012). Adrenocortical stem and progenitor cells: implications for adrenocortical carcinoma. Molecular and Cellular Endocrinology.

[bib53] Slater EP, Diehl SM, Langer P, Samans B, Ramaswamy A, Zielke A, Bartsch DK (2006). Analysis by cDNA microarrays of gene expression patterns of human adrenocortical tumors. European Journal of Endocrinology.

[bib54] Soon PS, Gill AJ, Benn DE, Clarkson A, Robinson BG, McDonald KL, Sidhu SB (2009). Microarray gene expression and immunohistochemistry analyses of adrenocortical tumors identify IGF2 and Ki-67 as useful in differentiating carcinomas from adenomas. Endocrine-Related Cancer.

[bib55] Staaf J, Vallon-Christersson J, Lindgren D, Juliusson G, Rosenquist R, Hoglund M, Borg A, Ringner M (2008). Normalization of Illumina Infinium whole-genome SNP data improves copy number estimates and allelic intensity ratios. BMC Bioinformatics.

[bib56] Takai D, Gonzales FA, Tsai YC, Thayer MJ, Jones PA (2001). Large scale mapping of methylcytosines in CTCF-binding sites in the human H19 promoter and aberrant hypomethylation in human bladder cancer. Human Molecular Genetics.

[bib57] Thompson LD (2002). Pheochromocytoma of the adrenal gland scaled score (PASS) to separate benign from malignant neoplasms: a clinicopathologic and immunophenotypic study of 100 cases. American Journal of Surgical Pathology.

[bib58] Tissier F, Cavard C, Groussin L, Perlemoine K, Fumey G, Hagnere AM, Rene-Corail F, Jullian E, Gicquel C, Bertagna X (2005). Mutations of β-catenin in adrenocortical tumors: activation of the Wnt signaling pathway is a frequent event in both benign and malignant adrenocortical tumors. Cancer Research.

[bib59] Tost J, Gut IG (2007). DNA methylation analysis by pyrosequencing. Nature Protocols.

[bib60] Ulaner GA, Yang Y, Hu JF, Li T, Vu TH, Hoffman AR (2003). CTCF binding at the insulin-like growth factor-II (IGF2)/H19 imprinting control region is insufficient to regulate IGF2/H19 expression in human tissues. Endocrinology.

[bib61] Van Loo P, Nordgard SH, Lingjaerde OC, Russnes HG, Rye IH, Sun W, Weigman VJ, Marynen P, Zetterberg A, Naume B (2010). Allele-specific copy number analysis of tumors. PNAS.

[bib62] Volante M, Buttigliero C, Greco E, Berruti A, Papotti M (2008). Pathological and molecular features of adrenocortical carcinoma: an update. Journal of Clinical Pathology.

[bib63] Voz ML, Mathys J, Hensen K, Pendeville H, Van Valckenborgh I, Van Huffel C, Chavez M, Van Damme B, De Moor B, Moreau Y (2004). Microarray screening for target genes of the proto-oncogene PLAG1. Oncogene.

[bib64] Waldmann J, Fendrich V, Holler J, Buchholz M, Heinmoller E, Langer P, Ramaswamy A, Samans B, Walz MK, Rothmund M (2010). Microarray analysis reveals differential expression of benign and malignant pheochromocytoma. Endocrine-Related Cancer.

[bib65] Weiss LM (1984). Comparative histologic study of 43 metastasizing and nonmetastasizing adrenocortical tumors. American Journal of Surgical Pathology.

